# Bioethical Issues as Triggers of Religious Transformation in Orthodox Christianity

**DOI:** 10.1111/bioe.70006

**Published:** 2025-06-15

**Authors:** Roman Tarabrin

**Affiliations:** ^1^ Faculty of Theology University of Fribourg Fribourg Switzerland; ^2^ Institute of Sociology Sechenov First Moscow State Medical University Moscow Russia

**Keywords:** basis of social concept, In Vitro fertilization, interaction of religious and biomedical worldviews, religious bioethics, Russian Orthodox Church

## Abstract

The advent of new biomedical technologies has given rise to an emerging area of sociocultural discourse. The sociocultural perception of these technologies is contingent upon a number of factors, including the prevailing attitudes within dominant religious traditions. Religious bioethics is fundamentally distinct from secular bioethics. The former is grounded in unchanging sacred scriptures and traditions, which inform its normative provisions. Consequently, a shift in the perception of technology must be accompanied by a corresponding shift in how religious institutions interpret scripture and tradition. This article employs the Russian Orthodox Church (ROC) as a case study to investigate how religious institutions can adapt to changing societal and cultural demands, and whether religious moral decrees can evolve in response to shifting sociocultural discourse. A discourse analysis of the ROC's interactions with the medical community and the general public reveals the following: To maintain influence with its followers, a religious institution should not categorically reject new advances in biomedicine. Rather, it should engage in a comprehensive bioethical analysis of the challenges posed by each emerging technology. In this process, it is valuable to define boundaries based on religious doctrine—limits that a believer must not exceed to maintain communion with the deity—while allowing for the use of new biomedical solutions.

## 1 Introduction

The social landscape is currently experiencing a period of significant transformation. The integration of novel biomedical technologies into sociocultural discourse is an emergent phenomenon that is gradually permeating the fabric of daily medical practice. These technologies are being progressively accepted by patients and are being perceived as ethically acceptable. Examples of such technologies include in vitro fertilization (IVF), the insertion of a sperm inside an egg (ICSI, or Intra Cytoplasmic Sperm Injection), vaccines based on genetic engineering, and genetic “scissors” technology (CRISPR‐Cas9), organ transplantation, among others. The sociocultural perception of these technologies is undergoing a corresponding transformation, which means that such technologies are becoming integrated into the fabric of people's lives, functioning as routine medical procedures and being employed by governments to prevent the spread of epidemics. To safeguard the interests of both the individual and humanity as a whole, bioethics has emerged as a discipline [1]. In the interdisciplinary field of bioethics, the potential risks and consequences of biotechnologies are evaluated through the application of universally established principles and rules. Consequently, with the emergence of a new procedure or technology, new legislative instruments are established to regulate the utilization of novel inventions [2].

Nevertheless, religious bioethics is fundamentally distinct from secular bioethics. For instance, the moral tenets of Christianity, along with its response to contemporary bioethical challenges, remain firmly rooted in unchanging biblical narratives, the decrees of the early Christian fathers, and the decrees of church councils [3, 4]. Consequently, there is a persistent quandary regarding nascent technologies: How can and should unchanging religious tenets respond to evolving societal acceptance of new technologies becoming a routine procedure? Conversely, will not the role of spirituality in the world be significantly undermined if religious bioethics fails to subject every technological innovation to critical scrutiny and unquestioningly endorses it?

This article examines the following: (1) What strategies can religious institutions adopt to respond effectively to the changing demands of contemporary society in its application to biomedical innovations? (2) Are religious moral decrees undergoing transformation in response to changing sociocultural perspectives on medical treatment? To answer these questions, we will examine the evolution of Orthodox Christian teachings on bioethical issues using the Russian Orthodox Church as a case study. The Russian Orthodox Church (ROC) was the first in the Orthodox world to propose a solution to bioethical issues in 2000 and has subsequently demonstrated the capacity for successful adaptation of its teachings.

Academic literature describes the activity of the ROC in response to the “social order for public ethics,” [5] which was expressed in the formulation of the “Basis of the Social Concept of the ROC” (BSC), demonstrating a shift in the position of the Church in relation to social issues [6]. Moreover, the studies indicate that the ROC has commenced reflection on novel bioethical matters [7]. However, the extant literature on the ROC addresses only those bioethical issues pertaining to the discourse on infertility, leaving unexamined the remainder of the issues and the Church's response to them. The contradiction in the ROC's social doctrine, which has resulted in significant religious confrontations, has once again been associated solely with the field of reproductive science [8, 9]. In light of the aforementioned considerations, this article will additionally address the following question: How can ambiguous religious doctrines influence the development of dilemmas within the society of believers, thereby becoming a catalyst for the transformation of a religious institution?

## 2 Materials and Methods

This article is based on discourse analysis of sociocultural debates in Orthodox Christian communities regarding biomedicine. It also examines the social doctrines and official statements of the Russian Orthodox Church in the context of biomedical research and bioethics.

## 3 Results

### 3.1 The Influence of Society on the Transformation of the Church

Despite the fact that the moral teachings of the Russian Orthodox Church are based on narratives and decrees from many centuries ago, in recent times the Church has been refining its doctrines in response to the demands of society. Already in the year 2000, the ROC had established its social doctrine, entitled “Basis of the Social Concept of the ROC” (BSC). One of the document's chapters is dedicated to the subject of bioethics. However, the document's bioethical provisions are, at times, contradictory. The current section of this article employs the example of bioethical dilemmas surrounding in vitro fertilization (IVF) to illustrate how such issues have contributed to the transformation of the ROC's bioethical position.

One of the initial controversies in Russian society to which the Church responded was the phenomenon of infertility and the appeal of in vitro fertilization for infertile couples. Among believers, just as among non‐believers, infertility has long been associated with suffering, leading to a high demand for recourse to reproductive technologies.^1^ The advent of technological advances, the proliferation of commercial clinics, and the integration of IVF within the ambit of compulsory medical insurance have collectively precipitated a societal shift in the perception of this medical intervention.^2^ The prevailing consensus in contemporary Russian society is that IVF constitutes a conventional medical procedure for the treatment of infertility.^3^

Nevertheless, certain groups in Russia, including Church representatives have asserted that, in accordance with the Orthodox worldview, IVF is wholly unacceptable [10]. A review of academic studies reveals that these groups have provided unbalanced coverage of Church documents without conducting sufficient analysis of bioethical issues within Orthodox discourse [4, 8]. As a consequence, the suffering of devout couples experiencing infertility has been intensified. Their condition has precluded them from having children of their own, and the Church's stance, as conveyed through these interpretations, has prohibited the utilization of accessible fertility treatments [11].

The initial effort to delineate the role of the religious institution in the resolution of this controversy concerning IVF can be observed in the synodal document (2013) entitled “On the Baptism of Infants Born with the Help of a Surrogate Mother.”^4^ Although the document primarily addresses the unacceptability of surrogate motherhood (SM), the ROC outlines its negative stance on SM by delineating the bioethical concerns with SM that are incongruent with Church teachings, rather than imposing a comprehensive prohibition on all reproductive technologies. Fr. Maxim Kozlov's^5^ commentary on this document substantiates the Church's acceptance of in vitro fertilization.^6^

Nevertheless, the lack of clarity in the ROC's social doctrine on IVF^7^ was employed by both proponents and opponents of reproductive technologies to reinforce their respective positions, which contributed to the fragmentation of the community of believers and further exacerbated the distress experienced by infertile Orthodox couples [12]. To clarify its position on the matter, the ROC decided to create a document analyzing the ethical problems of IVF and proposed ethically acceptable methods of IVF in accordance with the Orthodox worldview [13]. The document was distributed to dioceses and higher theological institutes, as well as being published on the official website of the ROC, to elicit feedback from clergy and laypeople. The Inter‐Council Presence of the ROC subsequently undertook an analysis of the feedback received, which revealed a multitude of contradictory positions on the published draft document.^8^

In light of the aforementioned sociocultural shifts towards IVF, the ROC's bioethical normative provisions underwent a transformation. The ROC initiated a discussion to elucidate its doctrinal tenets pertaining to the phenomenon of infertility, with the objective of maintaining its influence over its adherents. To this end, the religious institution employed a feedback mechanism to facilitate a comprehensive discourse on its normative provisions pertaining to the use of medical technology for the treatment of a serious ailment.

### 3.2 Other Bioethical Issues as the Potential for Changes in the Russian Orthodox Church

In Russia, the dichotomy of the religious institution's provisions regarding infertility and in vitro fertilization has resulted in a significant public controversy. Similarly, inaccuracies in other bioethical statements of any religious institution may provoke the increasing discrepancy between ecclesiastical doctrine and the sociocultural interpretation of biomedical issues. As a result, this may potentially weaken the role of religion in people's lives. Furthermore, individuals with religious convictions may encounter a dilemma when compelled to decide whether to adhere to their faith or seek medical counsel. Consequently, doctrinal statements formulated by religious institutions must be articulated with utmost clarity.

This section examines the institutional transformation of ROC (the formation of the Bioethical Committee), which emerged as a consequence of the endeavor to elucidate its bioethical provisions. The following three issues will be used to illustrate that even though the institutional transformation has occurred, ROC has to properly use its new department to clarify its bioethical statements and avoid unnecessary laypeople's suffering and their loss of faith.

Initially, the section introduces the issue that was successfully resolved by the ROC: the determination of the commencement of human life. Accordingly, it is hypothesized that this model should be employed to address all other bioethical issues. Then, the section elaborates on the example of vaccination against COVID to show that the resulting transformation of ROC may not function properly; therefore, it is essential to utilize it correctly. Particularly, to avoid ambiguities, bioethical provision of a religious institution has to deal with all the details of a biomedical innovation, including societal and religious consequences. And finally, the section points out the death criteria, which are obscured in ROC's documents and therefore have the potential to create divisions within the community of faith, as it happened with IVF, and therefore may and should trigger ROC to clarify its statement to strengthen adherence of its members.

#### 3.2.1 The Beginning of Human Life

Among the issues that have been successfully resolved are those pertaining to the moment of human emergence in the process of embryogenesis. With regard to the matter of intrauterine human development, the ROC has stated that abortion is “the gravest sin” [14]. The argumentation refers to canon law and sacred theological teachings that equate abortion with murder. However, as abortion is defined in medical terms as the termination of a pregnancy that has already commenced, the document in question makes no reference to the commencement of human life. This issue becomes pertinent when considering the possibility of terminating the life of embryos that have been cultivated outside the maternal body, for instance, in the context of IVF or for research purposes. It is inaccurate to state that an abortion occurs in the event of embryo death, given that pregnancy has not yet occurred. This, in turn, renders it impossible for the Church to condemn the destruction and modification of embryos obtained outside the mother's body.

A careful reading of the BSC reveals that the Church's stance on the origin of human life is even less clear. The procreation of the human being is considered a gift of God; thus, from the moment of conception, “any infringement on the life of the future human person is considered criminal” [14]. The reference to a “future person” introduces an element of ambiguity. The argument presented here does not assert that human life commences at the moment of conception, nor does it imply that abortion is inherently impermissible. Instead, the focus is on the infringement of the life of a “future person” [14]. There is a global debate regarding the juxtaposition of the human being as a member of a biological species, whose life truly begins at conception with the formation of a unique genetic code, and the human being as a person, a member of a moral community, whose life must be protected [15, 16]. In these discussions, the acceptance of the emergence of a “biological” human being at the moment of conception does not necessarily imply the acceptance of the emergence of a human person. Intentionally causing the death of a “biological” human being would not be considered murder, given that it is not yet a person. The BSC's assertion that the person is not a genuine person, but merely a future person, is pertinent to this debate and can be seen as supporting the idea of later emergence of a real person—a person who will emerge a little later than at the moment of conception.

The advancement of biomedicine and the subsequent utilization of its discoveries by religious groups has resulted in the necessity for further clarification of religious positions in order for ROC to maintain the religious adherence of its followers. The complexity of the debate surrounding conception and early embryonic development during IVF debates has shown the need for close collaboration with the scientific community. To this end, the ROC proactively engages in consultations with experts in reproductive medicine, biology, and the humanities during the drafting of documents on IVF and the beginning of human life. Additionally, roundtable discussions,^9^ some of which are webcast,^10^ and radio programs [17] featuring experts are held to facilitate informed discourse.

The necessity of maintaining close collaboration with the academic community, coupled with the need to address emerging scientific and technological challenges, underscores the imperative for a structural transformation of church institutions. In response, the Holy Synod has established the Synodal Commission on Bioethics, which, in addition to clergy, comprises scientists from a range of scientific disciplines. The principal objective of the Synodal Commission is to engage in discourse on bioethical matters “at a high theological and scientific level, with the participation of experts from a range of specialties and perspectives.”^11^

The Commission on Bioethics endorsed the Church document, “On the Inviolability of Human Life from the Moment of Conception,” which explicitly asserts that “human life commences at the moment of conception.”^12^ This argument informs the discourse on the inadmissibility of abortion and abortifacient contraception and may serve as a foundation for the Orthodox rejection of experimentation on embryos that are external to the maternal body, even though such experimentation does not align with the medical definition of abortion.

#### 3.2.2 Vaccination Against Coronavirus

The topic of vaccination in Orthodox discourse exemplifies the ROC's partial resolution of certain issues and illustrates the insufficient use of its new Bioethical Commission. During the outbreak of the novel coronavirus (2019‐nCoV), the Church confronted a new challenge related to biomedicine, necessitating an expedient resolution: the administration of vaccines against the 2019‐nCoV. Regrettably, the issues pertaining to vaccination had not previously been addressed in the BSC. Moreover, there is no recommendation for individual‐societal interactions, especially when vaccination is conducted with a pronounced paternalistic approach on the part of the state. This is why, in addition to other pandemic health‐related issues, believers had the following religious questions regarding the groundbreaking vaccine options: 1. Is it ethically permissible to refuse vaccination even when one's own life is threatened? 2. Is it ethically permissible for the state to mandate vaccination in the absence of extensive clinical trials for the vaccines in question? 3. Is it justifiable to exclude a healthy, unvaccinated believer from participating in religious rituals? And ultimately, 4. Would it be beneficial to ascertain the ethical acceptability within Orthodoxy of utilizing aborted tissue for the manufacture of vaccines?

In 2021, following the Russian government's creation and approval of several vaccines against the coronavirus infection, the ROC convened a roundtable discussion entitled “Vaccination: Ethical Aspects in light of Orthodox Faith,” on May 20, 2021. The discussions culminated in the formation of a final document. However, as this document was not officially approved by the Holy Synod or the Council of Bishops, it does not necessarily reflect the official position of the ROC. Instead, it represents the personal opinions of the church officials, priests, and scientists who participated in the discussion. It is regrettable that the Bioethical Commission had not yet been established at that time. Candidates for the commission were being actively discussed and prepared for approval by the Holy Synod. Consequently, only a limited number of priests and researchers were present at the deliberations concerning the pandemic and vaccination.

In light of the absence of reflection on vaccination issues in the BSC, it becomes evident that there is a need to formalize the position of church representatives, even if it is unofficial, on the subject of vaccination. In particular, the roundtable document on vaccination posits that compulsory vaccination cannot be justified by Orthodox teaching, as it is an individual decision. Each person must make their own choice regarding vaccination, based on their personal beliefs, knowledge, life experience, and information.^13^

A significant topic covered by the vaccination document is the utilization of embryonic tissues in vaccine production. The “Sputnik V” vaccine, the most prevalent in Russia, was developed using cells procured from aborted embryos. According to Orthodox doctrine, this constitutes a form of complicity in the death of an embryo [3, pp. 260–262]. The establishment of the Synodal Bioethical Commission in 2021 elevated this issue to a central point of contention within the ethical discourse surrounding vaccination, with the objective of formulating an official Church document.^14^ This prioritization is notable, as it occurs without any substantive discussion or resolution of other pressing societal and religious concerns, including issues such as coercion to vaccination, vaccine safety, and the exclusion of religious adherents from religious rituals.

It is understandable that the conditions of the pandemic precluded a full discussion of these issues. However, the fact that, after the end of the pandemic, they have not been formalized in the form of an official Church position raises concerns that they will be ignored in the future. Therefore, the initial transformation of the ROC as a religious institution in response to contemporary bioethical challenges may be used insufficiently and therefore significantly slow down the clarification of religious bioethical statements. This in turn, has the potential to evoke similar religious divisions that emerged in the context of in vitro fertilization.

#### 3.2.3 Death Criteria

Another set of biomedical issues that may give rise to divisions within the religious community pertains to the ethical considerations surrounding the end of human life. The BSC does not provide a clear definition of the criteria in Orthodoxy for determining human death. The determination of death is crucial for the removal of organs from the deceased, as the shorter the interval between death and organ removal, the more successful the organ transplantation is likely to be. However, the BSC does not provide definitive clarification regarding the criteria for human death as recognized by the ROC. The BSC asserts the necessity of eliminating “ambiguity in determining the moment of death” [14, section, XII, 7] with the objective of preventing the very procedure of organ removal from becoming the cause of death. Conversely, there is an obscure suggestion that a person remains alive only while functioning as a unified entity, which may indirectly indicate the Church's recognition of the brain‐based criteria for human death [14, XII, 8].

As in the case of IVF, the lack of clarity in the teaching of the ROC on end‐of‐life issues has resulted in contradictions within the Orthodox community, a lack of understanding of modern high‐tech procedures (in this case, transplantation), and a negative attitude towards such procedures among Orthodox Christians. In 2013, during the public discussion of the draft law “On the donation of organs, parts of human organs and their transplantation” in the ROC, a consolidated statement of synodal departments was published. This statement highlights the need to change the law from presumption of consent to presumption of dissent regarding posthumous removal of organs. It also makes a simultaneous call to parishioners to donate their organs. Nevertheless, the lack of understanding regarding the significance of this measure within the Orthodox community resulted in no increase in the number of donors. Furthermore, there remains a negative attitude toward the transplantation procedure itself and the issues surrounding it. To illustrate, one might cite the case of the defense of a master's thesis in one of the educational institutions of the Russian Orthodox Church. In this thesis, the student demonstrates that the ROC employs the brain criteria for determining human death, indicating that the Orthodox worldview allows for the removal of organs when the heart is still functioning [18]. The tenets of the thesis were met with a strongly adverse response. Concurrently, the academy members erroneously asserted that the ROC had resolved all transplantology issues and that the Church recognized the onset of human death solely upon the cessation of cardiac activity.

The bioethical issues under discussion pose a significant challenge for ROC, as societal acceptance of them may have a negative impact on individuals' adherence to religion. A comparable scenario may arise in various religious traditions globally. To maintain its adherents, a religious institution ought to elucidate its religious doctrine, taking into consideration their worldly existence.

## 4 Discussion: Is the Transformation of Religion Possible in Response to Emerging Challenges?

Biomedical advances can meet with two distinct responses from religious traditions. The initial response is one of complete rejection and prohibition, as religious moral teachings are based on unchanging sacred texts and tradition, which lack reflection on such issues. Those who espouse this viewpoint contend that since religious teachings do not offer guidance on the use of new technologies, there is no need to devise novel solutions, as any such innovations would be the product of the fallen human mind. This approach results in self‐isolation from the technologically advanced world and the limitation of religious followers' access to new technologies. This concept has been partially realized in the Russian context by the Public‐Church Council on Bioethics, which has maintained a unilateral stance on the inadmissibility of IVF for Orthodox individuals for over two decades [19].

Conversely, this article employs the example of the ROC to illustrate an alternative approach to distinguishing between ethically permissible and impermissible uses of biomedicine by religious individuals. The ROC has employed a meticulous bioethical examination of the challenges inherent in each emerging technology, delineating boundaries that a believer must not transgress to maintain communion with God. However, these boundaries also allow for personal freedom in determining whether or not to utilize novel biomedical solutions. Concurrently, the emerging trend of the Russian Orthodox Church indicates that freedom should be exercised with discernment. Those with greater spiritual maturity are advised to limit their use of civilization's achievements, whereas those who are newer to the faith are indulged, so to speak, and permitted to seek high‐tech assistance. The manner in which the Russian Orthodox Church has addressed the bioethical concerns surrounding IVF serves as an illustrative example of this approach. Rather than outright prohibition, the Church has sought to collaborate with the medical community in delineating the permissible limits of the utilization of assisted reproductive technologies for Orthodox believers [9].

Furthermore, the bioethical issues surrounding in vitro fertilization and the commencement of human life have served as a catalyst for the transformation of the Russian Orthodox Church in two significant ways. (1) The ROC opted to publicize a draft document for comprehensive debate among all believers, aiming to circumvent potential divisions within society. (2) The Holy Synod formed a specialized commission on bioethics to address these matters, thereby giving rise to structural alterations within the ROC. These transformations in the mode of discursive establishment of bioethical provisions have resulted in a shift from an authoritarian model to an authoritarian‐consultative model.

Also, they have presented a unique opportunity for a religious institution to initiate a previously unfeasible dialogue with the medical and scientific community. This dialogue could help alter public perceptions of the Church as an anachronistic and superfluous institution that impedes progress. Furthermore, the publication of draft documents, even in the absence of official approval, presents an opportunity to refute religious myths regarding the total prohibition of the use of new technologies.

Nonetheless, the institutional transformation may not function effectively, as evidenced in other cases. This may result in the inaccuracies in the bioethical provisions of the ROC remaining unresolved. The potential for such discourse to elicit discordance between religious doctrine concerning medicine and the sociocultural interpretation of bioethical issues was illustrated in the context of the IVF case. To circumvent unfavorable consequences, it is of the utmost importance that the ROC continue to engage in dialogue with professionals from the fields of medicine, biology, history, and sociology. This involvement would entail the ROC conducting a thorough bioethical analysis of each bioethical issue encountered by its adherents. By taking this approach, ROC can maintain its spiritual principles while aligning them with societal expectations regarding procedures, as evidenced by its response to infertility treatments.

## 5 Conclusion

Bioethical issues could become crucial in the formation of religious dilemmas among religious adherents. Individuals may encounter the decision whether to adhere to religious tenets or to pursue a medical procedure. This is why the articulation of religious moral decrees regarding biomedicine that are not clear and unambiguous has the potential to engender an increase in the moral and spiritual suffering of individuals, thereby weakening the role of religion in their lives. As demonstrated in our case study, the ambiguity in ROC's statements regarding in vitro fertilization has had a detrimental impact on the spiritual well‐being of infertile couples in Russia who adhere to religious beliefs.

In response to societal demands, the ROC initiated the process of elucidating its bioethical doctrine. In this process, the necessity of comprehensive communication with professionals in the sciences and humanities was recognized. However, this shift has necessitated a profound institutional transformation within the church, as evidenced by the establishment of a specialized committee on bioethics. This committee is responsible not only for its communication with academics but also with the general public. The organization has disseminated several documents to solicit public feedback on preliminary drafts. This phenomenon is indicative of not only the institutional transformation undergone by the ROC but also the new manner of establishment of normative statements. This method enables the church to sustain its connection with ordinary believers and articulate bioethical decisions that will be accepted by society.

Nevertheless, the consideration of societal demands does not imply an alteration of ROC's long‐standing decrees. The new bioethical committee is responsible for the critical task of aligning the church's spiritual tenets with the realm of biomedicine. In this capacity, the committee is tasked with establishing boundaries, or “red lines,” that delineate the ethical limits to which adherents of the church's teachings should adhere when engaging with the medical field. Consequently, the ROC has acknowledged the varying degrees of spiritual maturity among individuals and has granted them autonomy to determine the extent of their participation in novel biomedical procedures.

The integration of biomedical advances into the lives of the general public may result in a decline in the influence of religion. Nevertheless, the case of the ROC demonstrates that it is possible for a religious institution to support its members in their spiritual life and help them with their medical needs. It provides an illustration of how other religious institutions around the world might approach bioethical issues as they emerge over time. To this end, religious institutions should not eschew or prohibit the advancement of biomedicine outright. Instead, they should engage in a comprehensive bioethical analysis of emerging developments to ensure their alignment with religious tenets. This entails fostering open communication with the biomedical community, employing sociological and other methods of humanitarian research, and conducting extensive discourse on official documents. This process allows for the formulation of religious norms regarding biomedicine that can be accepted as legitimate by the entire religious community.

## Data Availability

Data sharing not applicable to this article as no datasets were generated or analyzed during the current study.

## Endnotes

^1^
As indicated in the report of the Russian Association of Human Reproduction, 1494 pregnancies were achieved in Russia through the use of IVF as early as 2000 [20].

^2^
In 2013, IVF was included in compulsory medical insurance [21].

^3^
In 2020, 39,743 IVF pregnancies were reported in Russia, representing a rate more than 20 times higher than in 2000 [22].

^4^
Holy Synod of the Russian Orthodox Church (Session December 25–26, 2013, No. 158) [23].

^5^
He currently serves as Chairman of the Educational Committee of the Russian Orthodox Church.

^6^
In particular, Archpriest Maxim Kozlov offers the following commentary on this document: “Concurrently, the Church does not prohibit IVF, except in the case of reduction, or the removal of supernumerary fertilized eggs” [24].

^7^
“Basis of social concept” of the ROC says that on the one hand, reproductive technologies are impermissible since such child conception is “disagreeable with the design of the Creator of life.” On the other hand, the same document allows to employ artificial insemination and states that IVF is prohibited if only it creates the excess number of embryos [25].

^8^
For a comprehensive examination of the aforementioned discussions, refer to Tarabrin [8].

^9^
Round Table “Ethical Problems Associated with the Method of In Vitro Fertilization,” November 23, 2019, All‐Church Postgraduate and Doctoral School, Moscow. Retrieved from http://www.doctorantura.ru/news/4256‐kruglyj‐stol‐eticheskie‐problemy‐svyazannye‐s‐metodom‐ekstrakorporalnogo‐oplodotvoreniya; International Roundtable “Bioethics Issues: A Christian Response,” May 11–14, 2021, Pyatigorsk, Russia. Retrieved from http://www.patriarchia.ru/db/text/5809271.html.

^10^
“Tsargrad” TV channel. Round table on “Ethical problems associated with the method of artificial reproduction,” 22.03.21. Retrieved from https://youtu.be/MEmtJvyzR_M.

^11^
Holy Synod of the Russian Orthodox Church (Session April 13, 2021). Retrieved from http://www.patriarchia.ru/db/text/5797581.html.

^12^
Document “On the Inviolability of Human Life from the Moment of Conception” In Holy Synod of the Russian Orthodox Church (Session December 27, 2023), 138. Retrieved from http://www.patriarchia.ru/db/text/6088088.html.

^13^
Final document of the round table “Vaccination: ethical aspects in the light of Orthodox Christianity” May 20, 2021, Moscow, Russia. Retrieved from http://www.patriarchia.ru/db/text/5811676.html.

^14^
It is noteworthy that the utilization of vaccines obtained through unethical means was previously addressed by the Orthodox Society of Russian Physicians in 2009. At that time, it was determined that the use of such vaccines was permissible in the absence of an alternative, given that there was no “deliberate killing of the fetus to obtain diploid cells” and “the use of vaccines produced from diploid cells had resulted in the saving of lives and the prevention of severe malformations in numerous individuals.” In Joint Statement of the Council and the Society of Orthodox Physicians of Russia on the Problems of Vaccination in Russia. Retrieved from http://www.patriarchia.ru/db/text/964218.html. Therefore, in the absence of a formal decision by the Synod of the Russian Orthodox Church on the use of fetal tissues for vaccine production, further discussion of this particular issue, which has already been informally addressed, is not necessary.
